# Impact of severe hypoglycemia on the heat shock and related protein response

**DOI:** 10.1038/s41598-021-96642-8

**Published:** 2021-08-23

**Authors:** Alexander S. Atkin, Abu Saleh Md Moin, Manjula Nandakumar, Ahmed Al-Qaissi, Thozhukat Sathyapalan, Stephen L. Atkin, Alexandra E. Butler

**Affiliations:** 1 Trinity College, Cambridge University, Cambridge, UK; 2 Diabetes Research Center (DRC), Qatar Biomedical Research Institute (QBRI), Hamad Bin Khalifa University (HBKU), Qatar Foundation (QF), PO Box 34110, Doha, Qatar; 3 Academic Endocrinology, Diabetes and Metabolism, Hull York Medical School, Hull, UK; 4 Leeds Medical School, Leeds, UK; 5 Royal College of Surgeons of Ireland, PO Box 15503, Adliya, Bahrain

**Keywords:** Type 2 diabetes, Diabetes complications

## Abstract

Heat shock proteins contribute to diabetes-induced complications and are affected by glycemic control. Our hypothesis was that hypoglycemia-induced heat shock and related protein changes would be amplified in type 2 diabetes (T2D). This prospective, case–control study enrolled 23 T2D patients and 23 control subjects who underwent hyperinsulinemic-induced hypoglycemia (≤ 2.0 mmol/L (36 mg/dl)) with blood sampling at baseline, at hypoglycemia and after a 24-h post-hypoglycemia follow-up period. Proteomic analysis of heat shock-related and pro-inflammatory proteins was performed. At baseline, MAPKAPK5 (*p* = 0.02) and UBE2G2 (*p* = 0.003) were elevated and STUB1 decreased (*p* = 0.007) in T2D. At hypoglycemia: PPP3CA (*p* < 0.03) was increased and EPHA2 (*p* = 0.01) reduced in T2D; by contrast, three proteins were reduced in controls [HSPA1A (*p* = 0.007), HSPB1 (*p* < 0.02), SMAD3 (*p* = 0.005)] while only MAPKAPK5 was elevated (*p* = 0.02). In the post-hypoglycemia follow-up period, most proteins normalized to baseline by 24-h; however, STIP1 (*p* = 0.003), UBE2N (*p* = 0.004) and UBE2L3 (*p* < 0.04) were decreased in controls at 24-h. No protein differed from baseline at 24-h in T2D. Pro-inflammatory interleukin-6 increased at 4-h post-hypoglycemia in controls and T2D (*p* < 0.05 and *p* < 0.003, respectively) and correlated with HSPA1A; anti-inflammatory IL-10 decreased 2-h post-hypoglycemia in T2D only. Other pro-inflammatory proteins, IL-1α, IFN-γ and TNF-α, were unchanged. Heat shock and related proteins differed at baseline between T2D and controls, with an exaggerated response of heat shock and related proteins to hypoglycemia that returned to baseline, though with changes at 24-h in controls alone. An increase in pro-inflammatory IL-6, with a decrease in anti-inflammatory IL-10, suggests that the HSP system is overactivated due to underlying inflammation in T2D.

Trial registration: ClinicalTrials.gov NCT03102801.

## Introduction

Type 2 diabetes (T2D) is characterized by chronic hyperglycemia secondary to increased insulin resistance (IR) in peripheral organs combined with progressive pancreatic islet β-cell failure^1^. T2D prevalence has reached pandemic proportions and is now the fourth leading cause of mortality^2^. Diabetes-related complications such as nephropathy, retinopathy, heart failure, limb amputation and stroke are difficult and costly to manage and place a significant burden on health care systems. Whilst the underlying pathophysiology of diabetes-related complications is increasingly understood, much more needs to be done to identify new molecular therapeutic targets. Diabetes has been considered to be a protein misfolding disease with islet amyloid polypeptide (IAPP) contributing to β-cell dysfunction and disease^3^. Heat shock proteins (HSPs) have a key role in protecting against protein misfolding through the ubiquitin proteasome system (UPS), the primary mechanism effecting degradation of short-lived, damaged or misfolded proteins^4^. Misfolded proteins contribute to the development and progression of T2D with accumulation of IAPP in islets (promoting dysfunction of beta cells) and other extra-pancreatic tissues^3^. The unfolded protein response (UPR) is of major importance in the cellular apparatus for clearance of short-lived, damaged and misfolded proteins. The degradation of such proteins is coordinated by the sequential action of three enzymes: ubiquitin-activating enzyme (E1), ubiquitin-conjugating enzyme (E2) and ubiquitin-protein ligase (E3). Following ubiquitination, proteolysis then proceeds through the 26S proteasome^5^. Consequently, HSPs have a predominantly cytoprotective role and downregulation of HSPs associates with dysfunctional insulin signaling^6^.

**Figure 1:**
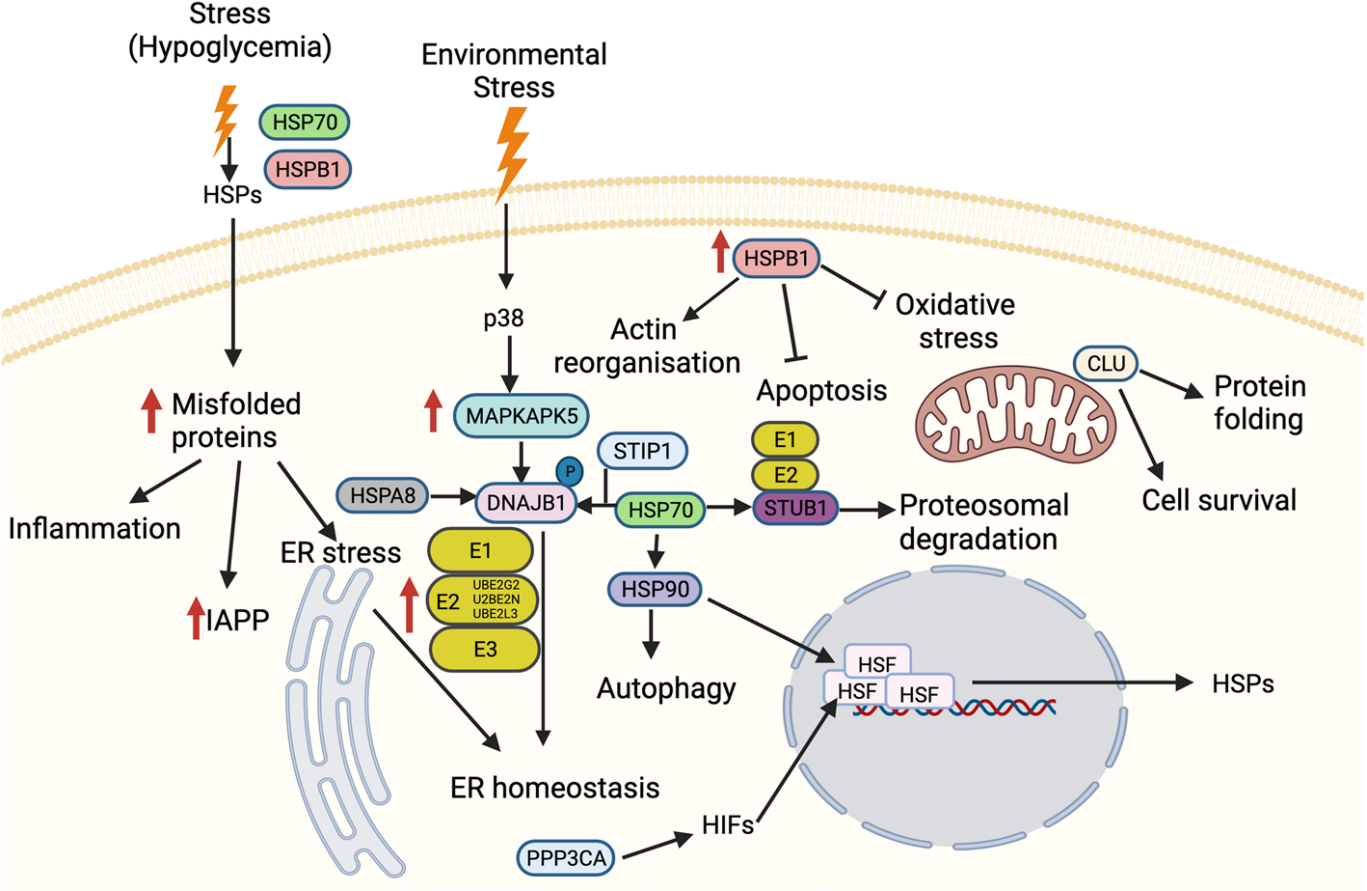
Schematic figure showing an overview of interactions between proteins involved in HSP and associated stress-response pathways in response to hypoglycemia. These interactions decide the fate of the downstream signaling pathway. The HSPs and associated proteins interact with the cell surface receptors and/or with each other in response to different stimuli, including accumulated unfolded/misfolded proteins, hormones and cellular/environmental stress (including hypoglycemia) (as indicated by upward red arrows) and regulate different molecules affecting a spectrum of biological functions such as apoptosis, autophagy, cell migration and alterations in the immune response. Schematic created using Biorender (https://biorender.com).

The heat shock response (HSR) is a cellular response that results in molecular chaperone expression to address the adverse effects on proteins due to endogenous and exogenous stressors such as temperature, oxidative stress, inflammation and heavy metals^7^. Whilst HSP and their associated proteins are constitutively expressed, they are rapidly upregulated by the cell stress response^7^. The HSR may be a global or a partial response, depending on the tissue affected and the nature and severity of the stress, with transient stress responses eliciting a differential protein response^8^. This leads to a complex and orchestrated response of the HSPs and their associated proteins and molecular chaperones that is shown in overview in Fig. 1 and detail in Supplementary Fig. S1. However, what specific stresses and/or their degree of stress that may elicit a global or partial HSR is still unknown.

HSPs are categorized by molecular weight^9^ and they take part in a wide array of cellular functions, physiological as well as stress-induced. Such functions include repression of protein aggregation, aiding folding and stability of newly-formed or damaged proteins, shuttling of proteins between cellular compartments and identifying irreversibly damaged proteins for degradation^10^. Recent evidence indicates HSP involvement in binding and controlling the activity of several critical enzymes involved in inflammation, apoptosis, metabolism and cell signalling^11^.

Genetic manipulation of certain HSPs, or modulation of their expression, has revealed their role in the pathogenesis of several chronic diseases including diabetes^12^. Acute hyperglycemia inhibited the protective upregulation of HSP32 and HSP70 in the liver of rats subjected to an ischemia/reperfusion injury following myocardial infarction and stroke^13^, though opposing findings have been reported in other organs, such as brain^14^ and kidney^15^, where HSPs are upregulated following a comparable insult. In patients with T2D, HSP90 has been reported to positively correlate with fasting blood glucose^16^. HSPs are also associated with diabetes-related complications: elevated HSP27 has been associated with diabetic neuropathy^17^ and diabetic nephropathy^18^. HSP70 has been associated with diabetes retinopathy^19^ and is inversely related to macrovascular complications^19^.

Optimal management of T2D dictates tighter glucose control with reduced glucose fluctuations, the drawback of such management being the increase in risk and frequency of hypoglycemic events. Hypoglycemia is linked de facto to detrimental sequelae, such as cognitive dysfunction and dementia^20^, and complicates management of these diabetic patients. Changes in HSP expression have been functionally related to hyperglycemia, suggesting that changes in glucose levels are the critical factor in their generation; therefore, our hypothesis proposed that changes in HSP and related protein levels would be augmented during/following hypoglycemia in patients with T2D, resulting in pro-inflammatory protein generation. The design of this study was intended to mimic such a hypoglycaemic event as would be experienced by a diabetic patient in practice^21^. The effect of hypoglycemia on levels has not previously been studied; therefore, HSP and related protein levels together with a pro-inflammatory protein panel were analysed following acute hyperinsulinemia-induced hypoglycemia in T2D patients and non-diabetic controls.

## Methods

### Study design

As has previously been described^22^, “this was a prospective parallel study performed in 46, T2D (n = 23) and control (n = 23), adult subjects at the Diabetes Centre at Hull Royal Infirmary from March 2017 to January 2018. All subjects were Caucasian, aged 40–70 years. The duration of diabetes was < 10 years and all T2D subjects were on a stable dose of medication (metformin, statin and/or angiotensin converting enzyme inhibitor/angiotensin receptor blocker) over the prior 3 months. For T2D patient inclusion, only metformin as anti-diabetic therapy was allowed; other inclusion criteria were HbA1c < 10% (86 mmol/mol), and no hypoglycemic unawareness or hypoglycemia within a 3-month period. In the control group, diabetes was excluded with an oral glucose tolerance test. All subjects had a body mass index (BMI) between 18 and 49 kg/m^2^, normal renal and hepatic biochemical indices and no prior history of cancer, nor any contraindication to insulin infusion to achieve hypoglycemia (ischemic heart disease, epilepsy, seizure history, drop attacks, history of adrenal insufficiency and treated hypothyroidism).

### Study participants

All participants had a medical history, clinical examination, routine blood tests and an electrocardiogram performed. A continuous insulin infusion was performed to induce hypoglycemia as previously detailed^21^ with blood samples taken at hypo (time 0), 30 min, 1-h, 2-h and 4-h post-hypoglycemia. After 4-h, participants were provided lunch and the T2D cohort were given their (morning) diabetes medications. Patients later took their evening medication as prescribed. Subjects reattended 24-h following the induction of hypoglycemia; patients withheld their medications until they completed the blood tests in the fasted state, after which breakfast was provided. Prior to discharge, blood glucose was checked using a glucose analyser (HemoCue glucose 201 +) to ensure normal levels, together with other vital signs.

All participants provided written informed consent. The trial was approved by the North West-Greater Manchester East Research Ethics Committee (REC number:16/NW/0518), registered at www.clinicaltrials.gov (NCT03102801) on 06/04/2017 and conducted according to the Declaration of Helsinki”.

### Insulin infusion

The insulin infusion was performed as previously detailed^21^. “Following an overnight fast, bilateral ante-cubital fossa indwelling cannulas were inserted 30–60 min prior to the commencement of the test (0830 h). To induce hypoglycemia, soluble intravenous insulin (Humulin S, Lilly, UK) was given in a pump starting at a dose of 2.5 mU/kg body weight/min with an increment of 2.5 mU/kg body weight/min every 15 min by hypoglycemic clamp^23^, until two readings of capillary blood glucose measured by a glucose analyser (HemoCue glucose 201 +) ≤ 2.2 mmol/L (< 40 mg/dl) or reading of ≤ 2.0 mmol/L (36 mg/dl)^23^. The blood sample schedule was timed subsequently in respect to the time point that hypoglycemia occurred. Following the identification of hypoglycemia, intravenous glucose was given in the form of 150 ml of 10% dextrose and a repeat blood glucose check was performed after 5 min if blood glucose was still < 4.0 mmol/L.” Comparison of plasma glucose levels at baseline, at hypoglycemia and post-hypoglycemia up to 24-h is shown in Supplementary Fig. S2.

### Biochemical markers

As previously described^22^, “blood samples were separated immediately by centrifugation at 2000 g for 15 min at 4 °C, and the aliquots were stored at − 80 °C, within 30-min of blood collection, until batch analysis. Fasting plasma glucose (FPG), total cholesterol, triglycerides, and high-density lipoprotein (HDL) cholesterol levels were measured enzymatically using a Beckman AU 5800 analyser (Beckman-Coulter, High Wycombe, UK).

### Slow Off-rate Modified Aptamer (SOMA)-scan assay

Slow Off-rate Modified Aptamer **(**SOMA)-scan technology offers significant advantages relative to other protein biomarker platforms in terms of cost, time, required sample size, multiplexing capability, dynamic range, and readout flexibility. Protein quantification was accomplished utilizing a Slow Off-rate Modified Aptamer (SOMAmer)-based protein array on an in-house Tecan Freedom EVO liquid handling system (Tecan Group, Maennedorf, Switzerland) utilizing buffers and SOMAmers from the SOMAscan HTS Assay 1.3 K plasma kit (SomaLogic, Boulder, CO) according to manufacturer’s instructions and as described previously^24–26^. The assay was performed in 96-well plates containing up to 85 plasma samples, 3 quality control and 5 calibrator plasma samples. In brief, EDTA plasma samples were diluted into bins of 40%, 1% and 0.05%, and the following assay steps undertaken: “1) binding – analytes and primer beads (PB)-SOMAmers (fully synthetic fluorophore-labeled SOMAmer coupled to a biotin moiety through a photocleavable linker) were equilibrated; 2) Catch 1—all analyte/SOMAmers complexes were immobilized on a streptavidin-substituted support. Washing steps removed proteins not stably bound to PB-SOMAmers and bound protein was biotinylated; 3) Cleave—long-wave ultraviolet light was applied to release analyte-SOMAmer complexes into the solution; 4) Catch II—analyte-SOMAmer complexes were selectively immobilized on streptavidin support via the introduced analyte-borne biotinylation. Further washing was continued to select against unspecific analyte/SOMAmer complexes; 5) Elution—Denaturation caused disruption of analyte-SOMAmer complexes. Released SOMAmers serve as surrogates for quantification of analyte concentrations; 6) Quantification—hybridization to custom arrays of SOMAmer-complementary oligonucleotides.

Normalization of raw intensities, hybridization, median signal and calibration signal were performed based on the standard samples included on each plate, as previously described^24^”^27^.

Version 3.1 of the SomaScan Assay was utilized specifically targeting heat shock and inflammatory proteins in the SomaScan panel. Timepoints were at baseline, at the point of hypoglycemia and at posthypoglycemia timepoints of 0.5, 1, 2, 4 and 24 h.

### Data processing and analysis

Initial Relative Fluorescent Units (RFUs) were obtained from microarray intensity images using the Agilent Feature Extraction Software (Agilent, Santa Clara, CA). Raw RFUs were normalized and calibrated using the software pipeline provided by SomaLogic. This included (a) microarray hybridization normalization based on spiked-in hybridization controls, (b) plate-specific intensity normalization, (c) median signal normalization, and (d) median calibrator scaling of single RFU intensities according to calibrator reference values. Samples with a high degree of hemolysis (Haptoglobin log RFU < 10) were excluded from the analysis.

Statistical analyses were performed on log_2_ RFU values using R version 3.5.2 (R Foundation for Statistical Computing, Vienna, Austria) including base R package. Data handling and differential protein expression were analyzed using the autonomics and limma^28^ packages. For differential protein analysis, we applied limma models containing contrasts between timepoints, as well as contrasts between healthy and patients with diabetes at single timepoints. In both models, blocking by patient ID was performed to account for random effects. Batch effect correction was performed by adding batch as a covariate to the model. Limma obtained P values were corrected using the Benjamini–Hochberg method^29^.

### Statistical analysis

There are no studies detailing the changes in HSP response to hypoglycemia on which to base a power calculation. Sample size for pilot studies has been reviewed by Birkett and Day^30^. They concluded that a minimum of 20 degrees-of-freedom was required to estimate effect size and variability. Hence, we needed to analyze samples from a minimum of 20 patients per group. Data trends were visually evaluated for each parameter and non-parametric tests were applied on data that violated the assumptions of normality when tested using the Kolmogorov–Smirnov Test. Comparison between groups was performed at each timepoint using Student’s t-test. A *p* value of < 0.05 was considered statistically significant. Within-group comparisons are as follows: changes from baseline, and from hypoglycemia, to each subsequent timepoint were compared using Student’s t-test. Pearson’s correlation test was used between HSP and proinflammatory proteins. The sample size was too small to adjust for baseline covariates. Statistical analysis of the data to create the graphs presented was performed using Graphpad Prism (San Diego, CA, USA).

### Ethics approval and consent to participate

The trial was approved by the North West-Greater Manchester East Research Ethics Committee (REC number: 16/NW/0518), registered at www.clinicaltrials.gov (NCT03102801) on 06/04/2017 and conducted according to the Declaration of Helsinki. All participants provided written informed consent.

### Consent for publication

All authors gave their consent for publication.

## Results

Demographic and clinical characteristics of the 46 study participants (23 T2D subjects, 23 controls)^21^ are shown in Supplementary table S1. Twenty-three HSP-related proteins were included in the panel for analysis (Supplementary table S2), in addition to the pro-inflammatory protein panel that included interleukin1-alpha (IL-1α), interleukin-6 (IL-6), interleukin-10 (IL-10), interleukin-12 (IL-12), interferon-gamma (IFNγ) and tumor necrosis factor-alpha (TNFα). The glucose response throughout the experimental time-course is shown in Supplementary Fig. S2. All of the HSP-related proteins changes from baseline to hypoglycemia and from baseline to 24-h for controls and for T2D are shown in Supplementary table S2.

**Figure 2:**
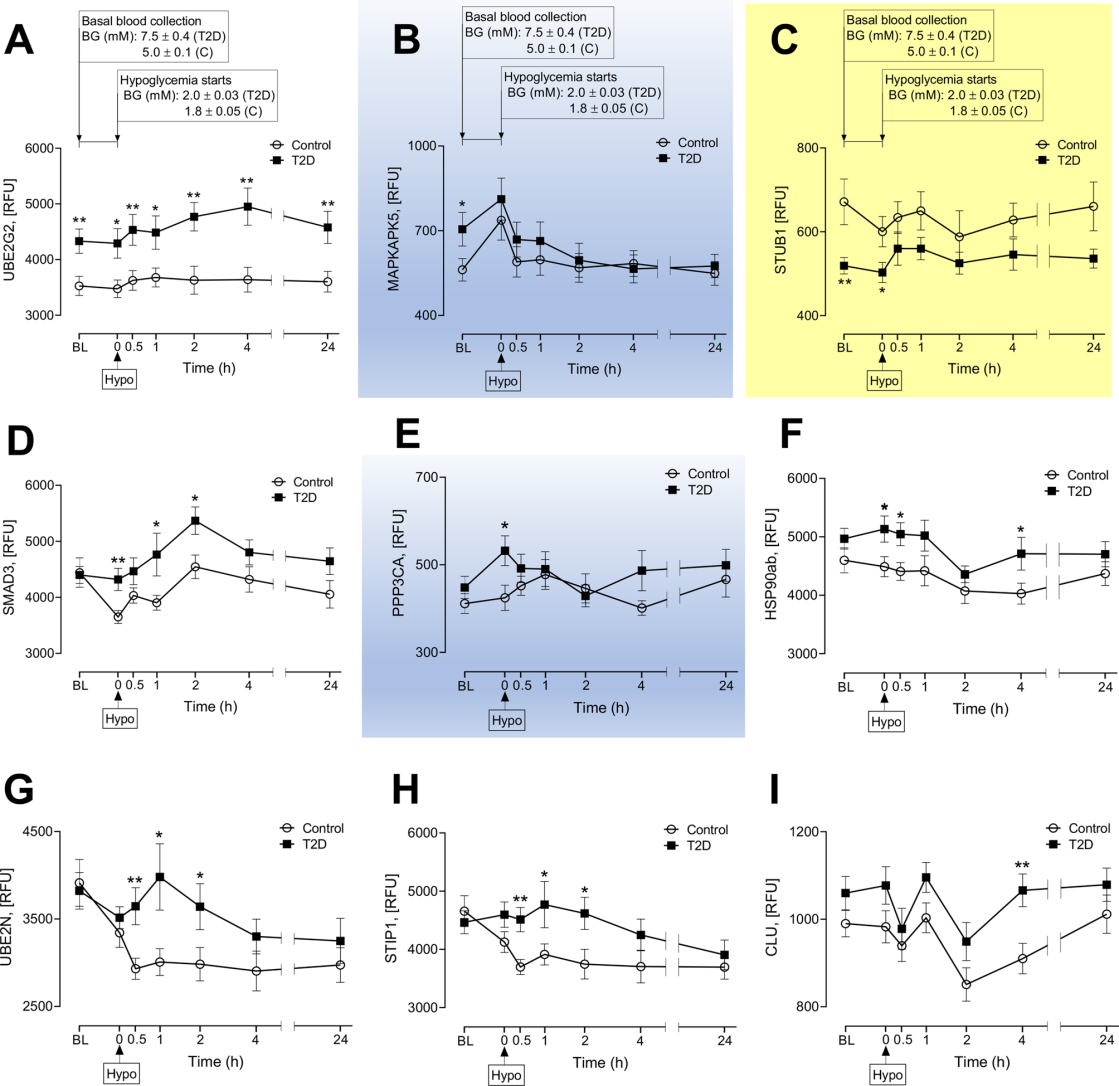
Comparison of circulatory heat shock protein (HSP) related proteins between control and T2D*.* Proteomic (Somalogic) analysis was undertaken to determine the plasma levels of heat shock proteins [HSPs], Ubiquitin-conjugating enzyme E2 G2 (UBE2G2) (**A**), MAP kinase-activated protein kinase 5 (MAPKAPK5) (**B**), E3 ubiquitin-protein ligase CHIP (STUB1) (**C**), Mothers against decapentaplegic homolog 3 (SMAD3) (**D**), Serine/threonine-protein phosphatase 2B catalytic subunit alpha isoform (PPP3CA) (**E**), Heat shock protein 90ab (HSP90ab) (**F**), Ubiquitin-conjugating enzyme E2 N (UBE2N) (**G**), Stress-induced-phosphoprotein 1 (STIP1) (**H**), Clusterin (CLU) (**I**) at baseline (BL), during and after iatrogenic induction of hypoglycemia for control (**C**) and type 2 diabetes (T2D) subjects. Blood sampling was performed at BL, at hypoglycemia (0 min) and post-hypoglycemia (0.5-h, 1-h, 2-h, 4-h and 24-h) for controls (white circles) and for T2D (black squares). Panels A-C show proteins for which levels differed at baseline between T2D and control subjects. Panels D-I show proteins for which levels differed at hypoglycemia and post-hypoglycemia between control and T2D. *p < 0.05, **p < 0.01, control vs T2D; RFU, relative fluorescent units; BG, blood glucose; Hypo, hypoglycemia. Those HSPs that generally show a trend to be consistently higher in T2D are not shaded; those HSPs that appeared to show no difference in trend between T2D and controls are shaded in blue; for the single HSP (STUB1) that is generally lower in T2D than controls, this graph is shaded in yellow.

**Figure 3:**
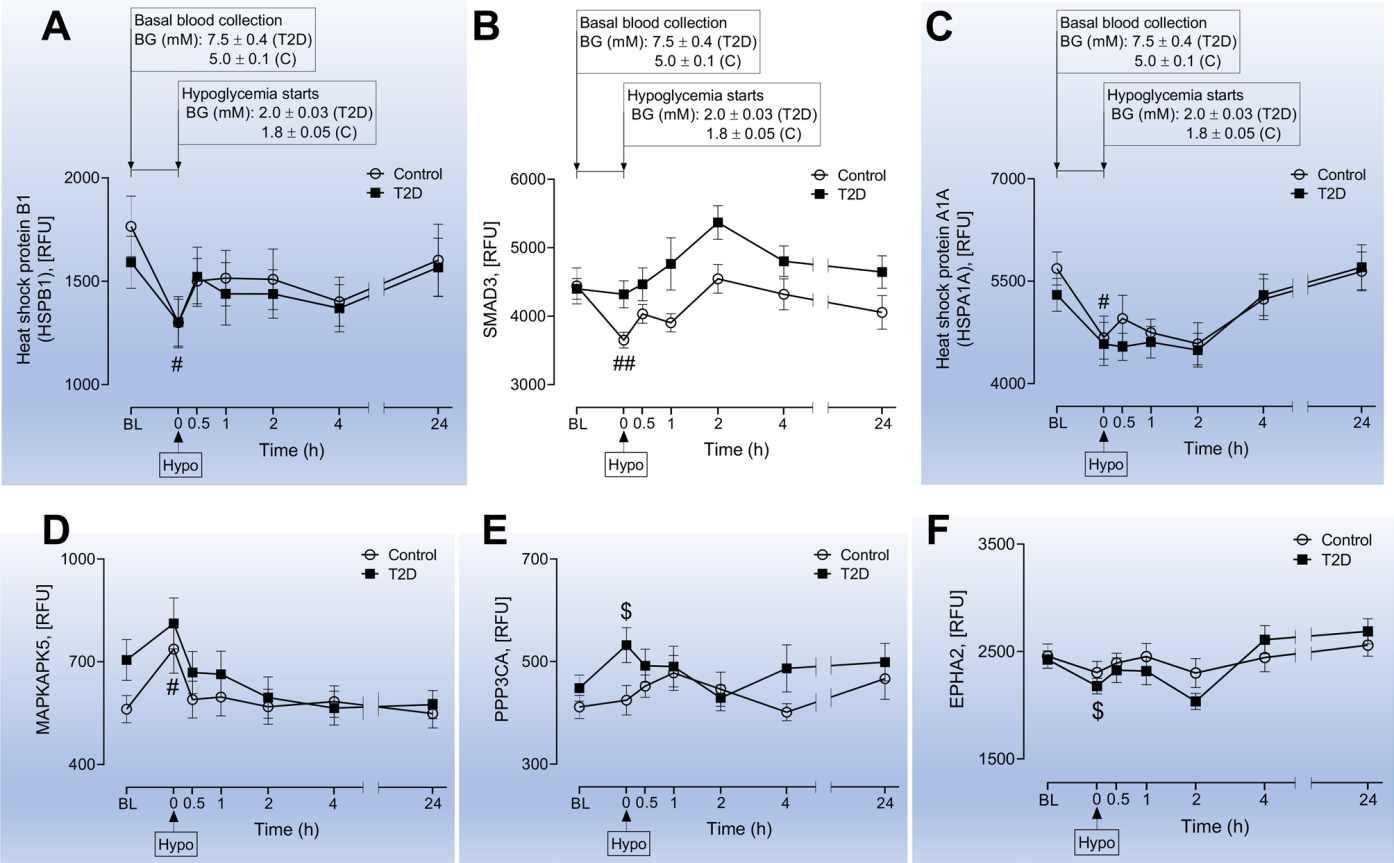
Comparison of circulatory HSP related proteins within groups (baseline vs hypoglycemia in Control subjects and subjects with T2D; significance is denoted by the symbol “#” for control and “$” for T2D). Proteomic (Somalogic) analysis was undertaken to determine the plasma levels of HSP related proteins, Heat shock protein beta-1 (HSPB1) (**A**), Mothers against decapentaplegic homolog 3 (SMAD3) (**B**), Heat shock 70 kDa protein 1A (HSPA1A) (**C**), MAP kinase-activated protein kinase 5 (MAPKAPK5) (**D**), Serine/threonine-protein phosphatase 2B catalytic subunit alpha isoform (PPP3CA) (**E**), Ephrin type-A receptor 2 (EPHA2) (**F**) at baseline (BL) during and after iatrogenic induction of hypoglycemia for control (C) and type 2 diabetes (T2D) subjects. Blood sampling was performed at BL, at hypoglycemia (0 min) and post-hypoglycemia (0.5-h, 1-h, 2-h, 4-h and 24-h) for controls (white circles) and for T2D (black squares). Panels A-D show proteins for which levels differed between baseline and hypoglycemia in control subjects (significance is denoted by “#”). Panels E–F show proteins for which levels differed between baseline and hypoglycemia in T2D subjects (significance is denoted by “$”). ^#^*p* < 0.05, ^##^*p* < 0.01, ^$^*p* < 0.05; RFU, relative fluorescent units; BG, blood glucose; Hypo, hypoglycemia. Those HSPs that generally show a trend to be consistently higher in T2D are not shaded; those HSPs that appeared to show no difference in trend between T2D and controls are shaded in blue.

**Figure 4:**
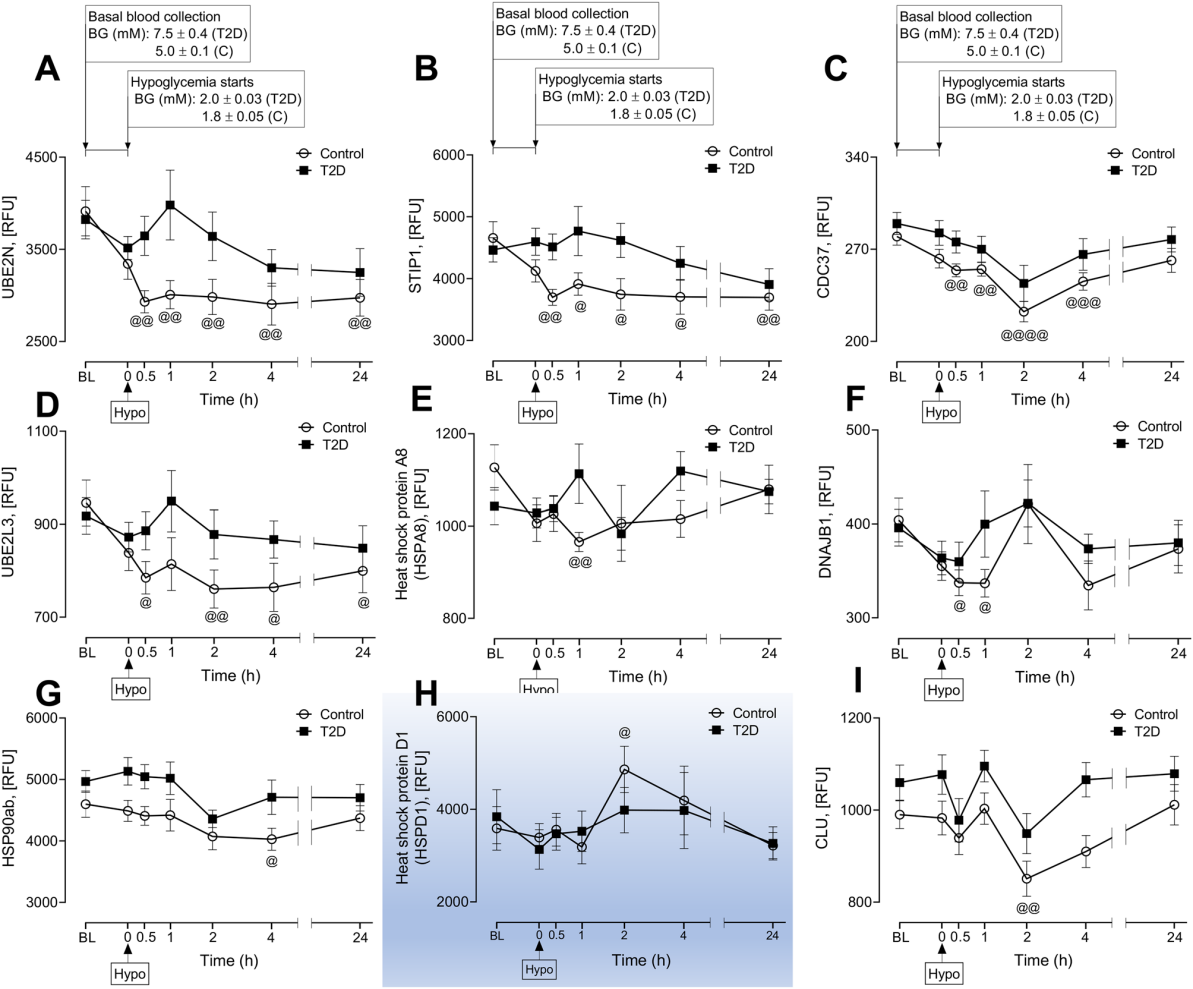
Comparison of circulatory HSP related proteins within groups at post-hypoglycemia (baseline vs 0.5–4 h post-hypoglycemia in control subjects; significance is denoted by the symbol “@”). Proteomic (Somalogic) analysis was undertaken to determine the plasma levels of HSP related proteins, Ubiquitin-conjugating enzyme E2 N (UBE2N) (**A**), Stress-induced-phosphoprotein 1 (STIP1) (**B**), Hsp90 co-chaperone Cdc37 (CDC37) (**C**), Ubiquitin-conjugating enzyme (UBE2L3) (**D**), Heat shock cognate 71 kDa protein (HSPA8) (**E**), DnaJ homolog subfamily B member 1 (DNAJB1) (**F**), Heat shock protein 90ab (HSP90ab) (**G**), 60 kDa heat shock protein, mitochondrial (HSPD1) (**H**), Clusterin (CLU) (**I**) at baseline (BL), during and after iatrogenic induction of hypoglycemia for control (C) and type 2 diabetes (T2D) subjects. Blood sampling was performed at BL, at hypoglycemia (0 min) and post-hypoglycemia (0.5-h, 1-h, 2-h, 4-h and 24-h) for controls (white circles) and for T2D (black squares). All the Panels from A-I show proteins for which levels differed between baseline and 0.5–4 h post-hypoglycemia in control subjects. ^@^*p* < 0.05, ^@@^*p* < 0.01, ^@@@^*p* < 0.001, ^@@@@^*p* < 0.0001; RFU, relative fluorescent units; BG, blood glucose; Hypo, hypoglycemia. Those HSPs that generally show a trend to be consistently higher in T2D are not shaded; those HSP that appeared to show no difference in trend between T2D and controls are shaded in blue.

**Figure 5:**
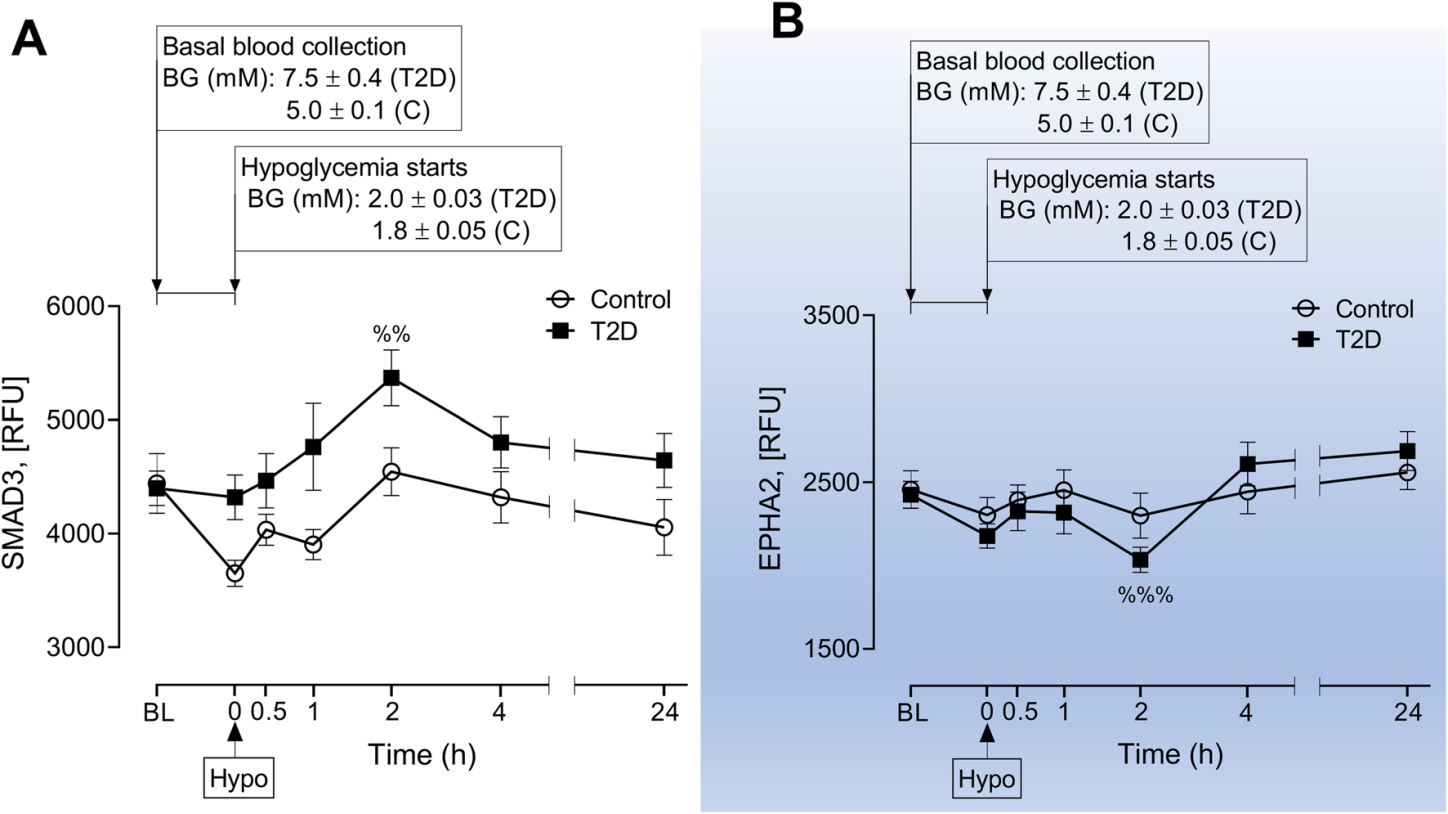
Comparison of HSP related proteins within groups at post-hypoglycemia (baseline vs 0.5–4 h post-hypoglycemia in subjects with T2D; significance is denoted by the symbol “%”). Proteomic (Somalogic) analysis was undertaken to determine the plasma levels of HSP related proteins, Mothers against decapentaplegic homolog 3 (SMAD3) (**A**), Ephrin type-A receptor 2 (EPHA2) (**B**). Blood sampling was performed at BL, at hypoglycemia (0 min) and post-hypoglycemia (0.5-h, 1-h, 2-h, 4-h and 24-h) for controls (white circles) and for T2D (black squares). Panels A-B show proteins for which levels differed between baseline and 0.5–4 h post-hypoglycemia in subjects with T2D. ^%%^*p* < 0.01, ^%%%^*p* < 0.001; RFU, relative fluorescent units; BG, blood glucose; Hypo, hypoglycemia. The HSP that generally shows a trend to be consistently higher in T2D (SMAD3) is not shaded; the HSP that appeared to show no difference in trend between T2D and controls (EPHA2) is shaded in blue.

Figures 2, 3, 4 and 5 show the differing analyses for the heat shock and related proteins over the time course for both T2D patients and controls that are detailed below. For those HSPs that generally show a suggested trend to be consistently higher in T2D in Figs. 2, 3, 4 and 5, these graphs are not shaded (CLU, SMAD3, HSP90ab, CDC37, HSPA8 (co-chaperone 70), STIP1, DNAJB1, UBE2L3, UBE2N, UBE2G2*)*; for the single HSP (STUB1) that is generally lower in T2D than controls, this graph is shaded in yellow; for those HSPs that appeared to show no difference in trend between T2D and controls (MAPKAPK5, PPP3CA, HSPB1, HSPA1A, EPHA2, HSPD1), these graphs are shown in blue.

### Differences between T2D and controls at baseline (significance is denoted by the symbol “*” in Fig. 2)

We first compared the heat shock and related proteins that differed at baseline between control subjects and subjects with T2D. At baseline, the levels of UBE2G2 and MAPKAP5 were elevated (4399.1 ± 152.8 vs 3525.3 ± 172.6 RFU of UBE2G2, T2D vs control, *p* < 0.01 and 705.1 ± 58.1 vs 561.6 ± 40.0, RFU of MAPKAP5, *p* < 0.05,) and the level of STUB1 was lower (519.02 ± 19.4 vs 671.4 ± 54.6 RFU of STUB1, T2D vs control, *p* < 0.05) in the T2D cohort (Fig. 2A–C).

### Differences between T2D and control at hypoglycemia (significance is denoted by the symbol “*” in Fig. 2)

UBE2G2 (Fig. 2A), SMAD3 (Fig. 2D), PPP3CA (Fig. 2E) and HSP90ab (Fig. 2F) was higher (4291.4 ± 264.9 vs 3476.4 ± 158.3 RFU of UBE2G2 at hypoglycemia, T2D vs control, *p* < 0.05; 4320.0 ± 196.4 vs 3650.4 ± 116.1 RFU of SMAD3 at hypoglycemia, T2D vs control, *p* < 0.05; 532.1 ± 34.1 vs 424.6 ± 29.0 RFU of PPP3CA at hypoglycemia, T2D vs control, *p* < 0.05; 5133.09 ± 224.5 vs 4489.3 ± 215.0 RFU of HSP90ab at hypoglycemia, T2D vs control, *p* < 0.05) and STUB1 was lower (Fig. 2C) (502.9 ± 24.2 vs 707.8 ± 112.9 RFU of STUB1 at hypoglycemia, T2D vs control, *p* < 0.05) at hypoglycemia in T2D compared to control.

### Differences between T2D and control after post-hypoglycemia (significance is denoted by the symbol “*” in Fig. 2)

UBE2G2 was elevated (Fig. 2A) all throughout post-hypoglycemia (0.5-h to 24 h) in T2D vs control (4534.2 ± 279.2 vs 3625.6 ± 176.8, 0.5-h post-hypoglycemia, *p* < 0.01; 4481.6 ± 287.3 vs 3613.9 ± 174.7, 1 h post-hypoglycemia, *p* < 0.05; 4768.6 ± 255.3 vs 3629.5 ± 252.0, 2 h post-hypoglycemia, *p* < 0.01; 4950.3 ± 334.7 vs 3638.3 ± 221.8, 4 h post-hypoglycemia, *p* < 0.01; 4578 ± 288.3 vs 3589.7 ± 188.7, 24 h post-hypoglycemia, *p* < 0.01). UBE2N (Fig. 2G) and STIP1 (Fig. 2H) were higher from 0.5-h to 2 h post-hypoglycemia in T2D compared to control. UBE2N (RFU): 3645.6 ± 211.2 vs 2931.4 ± 121.5, 0.5-h post-hypoglycemia, *p* < 0.05; 3925.6 ± 365.8 vs 2973.2 ± 151.4, 1 h post-hypoglycemia, *p* < 0.01; 3640.5 ± 263.8 vs 2983.3 ± 190.4, 2 h post-hypoglycemia, *p* < 0.01). STIP1 (RFU): 4512.7 ± 208.3 vs 3698.0 ± 129.5, 0.5-h post-hypoglycemia, *p* < 0.01; 4709.9 ± 384.8 vs 3858.5 ± 182.1, 1 h post-hypoglycemia, *p* < 0.05; 4618.0 ± 277.2 vs 3745.7 ± 255.6, 2 h post-hypoglycemia, *p* < 0.05. SMAD3 was higher at 1 h and 2 h post-hypoglycemia in T2D compared to control (4696.5 ± 371.1 vs 3870.1 ± 132.0, 1 h post-hypoglycemia, *p* < 0.05; 5369.6 ± 245.5 vs 4545.1 ± 211, 2 h post-hypoglycemia, *p* < 0.05). While HSP90ab was higher at 0.5 and 4 h post-hypoglycemia (Fig. 2F), CLU was higher only at 4 h posthypoglycemia (Fig. 2I) in T2D compared to control (5045.2 ± 196.7 vs 4408.2 ± 153.3 RFU of HSP90ab, 0.5-h post-hypoglycemia, *p* < 0.05 and 4711.8 ± 280.3 vs 4028.6 ± 179.4 RFU of HSP90ab at 4 h post-hypoglycemia, T2D vs control, *p* < 0.05; 1066.1 ± 12.0 vs 910.1 ± 34.8 RFU of CLU at 4 h post-hypoglycemia, T2D vs control, *p* < 0.01.

### Comparison of HSP related proteins within groups (hypoglycemia vs baseline in Control subjects, significance is denoted by the symbol “#” in Fig. 3)

We first compared plasma heat shock and related protein levels in response to transient hypoglycemia in control subjects. HSPB1 (Fig. 3A), SMAD3 (Fig. 3B) and HSPA1A (Fig. 3C) decreased significantly (1300.0 ± 113.7 vs 1765.6 ± 146.7 RFU of HSPB1, hypoglycemia vs baseline, *p* < 0.05, 3650.4 ± 116.1 vs 4443.5 ± 264.5 RFU of SMAD3, hypoglycemia vs baseline, *p* < 0.01 and 4673.1 ± 316.5 vs 5685.5 ± 242.1 RFU of HSPA1A hypoglycemia vs baseline in control, *p* < 0.05), whilst MAPKAP5 increased significantly (737.2 ± 70.6 vs 561.6 ± 40.0 RFU of MAPKAP5, hypoglycemia vs baseline in control, *p* < 0.05) in controls from baseline to hypoglycemia (Fig. 3D).

### Comparison of HSP related proteins within groups (baseline vs hypoglycemia in subjects with T2D, significance is denoted by the symbol “$” in Fig. 3)

In T2D, the level of PPP3CA was increased (532.1 ± 34.1 vs 448.3 ± 25.4 RFU of PPP3CA, hypoglycemia vs baseline, *p* < 0.05 (Fig. 3E) and the level of EPHA2 was decreased (2178.0 ± 73.0 vs 2425.1 80.0 RFU of EPHA2, hypoglycemia vs baseline, *p* < 0.05) (Fig. 3F) from baseline to hypoglycemia in T2D, though both returned to baseline values 0.5-h after the hypoglycemic episode.

### Comparison of HSP related proteins within groups at post-hypoglycemia (baseline vs 0.5–4 h post-hypoglycemia in control subjects, significance is denoted by the symbol “@” in Fig. 4)

We compared the HSP *related protein* levels between baseline versus post-hypoglycemic timepoints from 0.5-h to 4-h post-hypoglycemia. In control, ubiquitin conjugating enzymes UBE2N (Fig. 4A), stress-induced phosphor protein 1 (STIP1) (Fig. 4B) and co-chaperone CDC37 (Fig. 4C) were significantly decreased below baseline from 0.5 to 4-h [UBE2N (RFU): (2931.4 ± 121.4 vs 3912.5 ± 267.2, 0.5-h post-hypoglycemia vs baseline, *p* < 0.01; 2973.2 ± 151.4 vs 3912.5 ± 267.2, 1-h post-hypoglycemia vs baseline, *p* < 0.01; 2983.3 ± 190.4 vs 3912.5 ± 267.2, 2-h post-hypoglycemia vs baseline, *p* < 0.01; 2905.3 ± 227.2 vs 3912.5 ± 267.2, 4-h post-hypoglycemia vs baseline, *p* < 0.01. STIP1 (RFU): 3698.0 ± 129.5 vs 4656.4 ± 265.04, 0.5-h post-hypoglycemia vs baseline, *p* < 0.01; 3858.5 ± 182.1 vs 4656.4 ± 265.04, 1-h post-hypoglycemia vs baseline, *p* < 0.01; 3745.7 ± 255.6 vs 4656.4 ± 265.04, 2-h post-hypoglycemia vs baseline, *p* < 0.01; 3705.2 ± 280.8 vs 4656.4 ± 265.04, 4-h post-hypoglycemia vs baseline, *p* < 0.01. CDC37 (RFU): 254.0 ± 4.8 vs 279.6 ± 6.55, 0.5-h post-hypoglycemia vs baseline, *p* < 0.01; 255.1 ± 5.0 vs 279.6 ± 6.55, 1-h post-hypoglycemia vs baseline, *p* < 0.01; 222.5 ± 7.7 vs 279.6 ± 6.55, 2-h post-hypoglycemia vs baseline, *p* < 0.01; 245.5 ± 6.7 vs 279.6 ± 6.55, 4-h post-hypoglycemia vs baseline. Another ubiquitin enzyme UBE2L3 was also decreased from 0.5-h to 4-h post-hypoglycemia (Fig. 4D), however, the significant differences were observed at 0.5-h, 2-h and 4-h [UBE2L3 (RFU): (784.9 ± 35.1 vs 945.6 ± 49.3 0.5-h post-hypoglycemia vs baseline; 760.5 ± 40.8 vs 945.6 ± 49.3 2-h post-hypoglycemia vs baseline, *p* < 0.01; 760.0 ± 52.0 vs 945.6 ± 49.3, 4-h post-hypoglycemia vs baseline, *p* < 0.01].

When compared to baseline, plasma levels of some other HSPs also differed between 0.5 and 2-h post-hypoglycemia. For example, HSPA8 was lower at 1-h (988.2 ± 29.8 vs 1127.0 ± 49.1 RFU of HSPA8, 1-h post-hypoglycemia vs baseline, *p* < 0.01) (Fig. 4E); DNAJB1 was lower at 0.5 and 1-h (337.4 ± 13.7 vs 404.2 ± 23.2 RFU of DNAJB1, 0.5-h post-hypoglycemia vs baseline, *p* < 0.05; 338.8 ± 14.4 vs 404.2 ± 23.2 RFU of DNAJB1, 1-h post-hypoglycemia vs baseline, *p* < 0.05 (Fig. 4F). The levels of few proteins only differed at certain post-hypoglycemic timepoint. For example, HSP90ab was lower at 4-h post-hypoglycemia (4028 ± 179.4 vs 4598.4 ± 215.0, RFU of HSP90ab, 4-h post-hypoglycemia vs baseline, *p* < 0.05) (Fig. 4G); HSPD1 was higher at 2-h post-hypoglycemia (4863.2 ± 502.2 vs 3587.8 ± 471.1 RFU of HSPD1, 2-h post-hypoglycemia vs baseline, *p* < 0.05) (Fig. 4H); and CLU was lower at 2-h (851.0 vs 38.0 vs 990.0 ± 35.0 RFU of CLU, 2-h post-hypoglycemia vs baseline, *p* < 0.01) (Fig. 4I).

### Comparison of HSP related proteins within groups at post-hypoglycemia (baseline vs 0.5–4 h post-hypoglycemia in subjects with T2D, significance is denoted by the symbol “%” in Fig. 5)

In T2D, following hypoglycemia, SMAD3 was higher than baseline at 2-h (5369.6 ± 245.5 vs 4399.1 ± 152.8 RFU of SMAD3, 2-h post-hypoglycemia vs baseline, *p* < 0.01) (Fig. 5A). EPHA2 was lower than baseline at 2 h (2035.4 ± 76.0 vs 2425.1 ± 80.0 RFU of EPHA2, 2-h post-hypoglycemia vs baseline, *p* < 0.001) (Fig. 5B).

### Baseline to 24-h post-hypoglycemia in both T2D and controls (significance in control is denoted by the symbol “@” in Fig. 4)

At 24-h, UBE2N (2927.0 ± 206.3 vs 3912.5 ± 267.2 RFU of UBE2N, 24-h post-hypoglycemia vs baseline, *p* < 0.01, Fig. 4A); STIP1 (3645.2 ± 211.9 vs 4656.4 ± 265.04, 24-h post-hypoglycemia vs baseline, *p* < 0.01, Fig. 4B) and UBE2L3 (788.3 ± 47.8 vs 945.6 ± 49.3 RFU of UBE2L3, 24-h post-hypoglycemia vs baseline, *p* < 0.04) (Fig. 4D) were decreased in controls, a change that was not seen for those with T2D.

HSP and related proteins that did not differ with hypoglycemia or between T2D and controls included TLR-4, UCHL1, HSP90AA1 and CD274, and these are shown in Supplementary Fig. S3.

### Pro-inflammatory and anti-inflammatory protein changes

The pro-inflammatory cytokine, IL-6, increased at 4-h post-hypoglycemia in controls and T2D (*p* < 0.05 and *p* < 0.003, baseline vs 4-h, respectively) whilst the anti-inflammatory cytokine, IL-10, decreased 2-h post-hypoglycemia in T2D (*p* = 0.0001, baseline vs 2-h). Other cytokines, namely IL-1α, IL-12, IFNγ and TNFα, were unchanged (Supplementary Fig. S4A–F). HSPA1A (HSP70) correlated with IL-6 only in the control subjects (r = 0.47, *p* < 0.03) (Supplementary Fig. S4G, H).

## Discussion

Review of the overall trends of the heat shock response and related proteins in response to hypoglycemia in T2D and controls showed there were a group of heat shock and related proteins that, overall, tended to be consistently higher in T2D compared to controls (CLU, SMAD3, HSP90ab, CDC37, HSPA8 (co-chaperone 70), STIP1, DNAJB1, UBE2L3, UBE2N, UBE2G2*)*; only 1 protein was consistently lower in T2D (STUB1) whilst a group of heat shock and related proteins appeared to show no difference in trend between T2D and controls (MAPKAPK5, PPP3CA, HSPB1, HSPA1A, EPHA2, HSPD1). Despite a relatively short duration of disease in our T2D cohort, clear differences were observed between control and T2D subjects in their heat shock and related protein response, with the suggestion that many of the heat shock and related proteins were higher in T2D over the time course. This suggests that these heat shock and related proteins were either constitutively activated or preconditioned for an enhanced response of the heat shock and related proteins in T2D; however, only UBEG2 and MAPKAP5 differed significantly at baseline between control and T2D subjects. Many of the subsequent changes noted following hypoglycemia were seen in the controls and not in T2D subjects, perhaps suggesting that the overactivation of the heat shock and related protein response in T2D may be maximally protective and may not have the capacity to incrementally increase in response to the additional stress from the hypoglycemic event. If the heat shock and related protein defenses are already maximally stimulated, then the additional stress of hypoglycemia may overwhelm this defense mechanism, leading to a greater chance of misfolded proteins causing damage and leading to diabetes complications. Recurrent hypoglycemic episodes may therefore lead to the development, or the promotion, of diabetes-related complications and, together with the evidence that hyperglycemia may adversely affect HSPs^13–16^, this combination of prevailing hyperglycemia together with hypoglycemic episodes in the setting of diabetes may lead to the acceleration of complication development.

For those heat shock and related proteins that appeared to trend higher in the time course in T2D compared to controls, that included CLU, SMAD3, CDC37, HSPA8 (co-chaperone 70), DNAJB1, STIP1, HSP90ab, UBE2L3, UBE2N and UBE2G2*,* it may be seen in Fig. 1 and Supplementary Fig. S1 that HSPA8 (co-chaperone 70), DNAJB1, STIP1, HSP90ab, UBE2L3, UBE2N, UBE2G2 are integrally linked and therefore this entire pathway may be increased in T2D. For example, DNAJB1 functions as a co-chaperone with HSPA1A (HSP70) and promotes protein handling (folding, transport and degradation), acting through UBE2G2 as noted above^31^. Exogenous insulin may have stimulated SMAD3 that is the main signal transducer for transforming growth factor beta that regulates insulin gene transcription, with higher levels of SMAD3 suppressing insulin content^32^. CLU overexpression may be seen to be protective for apoptosis and may stimulate HSP90, that also tended to be increased in T2D^33^.

There are no paradigms of heat shock and related protein overactivation or of a preconditioned heat shock and related protein response to stress in T2D, therefore these observations are novel. However, it is unknown whether this heat shock and related protein overactivation would cause specific defects in cellular function that may impact upon diabetes or upon the effect of hypoglycemia other than, as noted above, that an additional stress insult would overwhelm the heat shock and related protein system leading to misfolding and the promotion of complications. Whilst analyzing the protein levels in the plasma in the current study, it is difficult to determine whether the unfolded protein response (UPR) is overactivated in certain tissues in T2D. However, studies in other systems have demonstrated that overactivation of the UPR in brain tissue led to neurodegenerative diseases. For example, Moreno and colleagues^34^ provided the first demonstration that chronic PERK signaling is detrimental to neuronal survival and drives neurodegeneration in prion-diseased mice; the authors reported that the accumulation of misfolded Prion proteins (PrP) during disease leads to chronically elevated levels of PERK-P and eIF2a-P, resulting in the sustained reduction in global protein synthesis rates in the brain.

STUB1 was persistently lower in T2D compared to controls but it is unclear why this should be the case given that it interacts with HSP90ab that was elevated. STUB1 is an E3 ubiquitin ligase that is chaperone-dependent and interacts with HSPA1A (HSP70) and HSP90 (HSP90AA1/AB1) to mediate the ubiquitination and proteasomal degradation of receptors, such as Toll-like receptor 4^35^, and its decrease has been associated with enhanced oxidative stress cell survival^36^. One potential mechanism for the decrease in STUB1 is based upon a previous report demonstrating that stress due to hyperglycemia decreases the ubiquitin E3 ligase activity of Mdm2 by inducing the phosphorylation of this residue in RINm5F cells^37^; therefore, it is likely that hyperglycemia due to insulin resistance may cause lower levels of STUB1 in T2D.

For those heat shock and related proteins that appeared to not be differentially expressed in response to hypoglycemia, MAPKAPK5 may be stimulated by environmental factors^38^ and interacts with HSPB1 and is therefore likely to be unrelated to hypoglycemia^39^. Unlikely to be affected by hypoglycemia, PP3CA has an essential role in the transduction of intracellular calcium^40^ but appeared not to differ between T2D and controls. HSPA1A (HSP70) is important to many of the HSP responses shown in Fig. 1 and Supplementary Fig. S1, and any dysfunction may have critical effects; therefore, it is not surprising that there was no difference between T2D and controls. HSPD1 is a signaling molecule in the innate immune system^41^ and is therefore less likely to be affected by hypoglycemia.

In control subjects, HSPB1, SMAD3 and HSPA1A decreased significantly, whilst MAPKAPK5 increased significantly in controls from baseline to hypoglycemia whilst, in T2D, PPP3CA increased and EPHA2 decreased. Visually both groups appeared to parallel each other, though that significance was not achieved between baseline and hypoglycemia in the T2D group may be a power issue.

Notably UBE2N, UBE2L3, and STIP1 all significantly go down in controls but not at all in T2D in response to hypoglycemia, whilst the protein that is linked within the system, UBE2G2, was unaffected by hypoglycemia, though significantly lower than levels seen in T2D at all time points.

The differing roles of HSPs in diabetes is evolving. HSPB1 has been shown to be elevated in T2D patients with diabetic nephropathy^9^ and it is highly expressed in podocytes and increased in vitro by high glucose; it is thought that HSPB1 is likely to have a protective effect to limit apoptosis and oxidative stress^42^; HSPB1 is reported to be overexpressed in diabetic retinopathy^43^. Endoplasmic reticulum (ER) stress is a consequence of hyperglycemia and is important in development of diabetic retinopathy, though the exact mechanism leading to damage is unclear. UBE2G2 is a key player in the ubiquitin proteasome system that is important in restoring ER homeostasis and maintaining normal ER function^44^.

Glucose variability may affect HSPs in T2D, so increasing their response in hypoglycemia^45^, and hypoglycemia has been reported to induce cellular stress in different in vitro studies. For example, Kato et. al., showed that recurrent short-term hypoglycemia and hyperglycemia induce apoptosis and oxidative stress via the ER stress response in immortalized mouse Schwann (IMS32) cells^46^; however, any direct link between hypoglycemia and tissue specific protein misfolding in humans with T2D has not yet been reported. It is therefore unlikely that there is a protective cellular response to misfolding specifically because of hypoglycemia, but rather a response to the cellular stress that results from hypoglycemia.

In the time course following hypoglycemia, UBE2N, UBE2L3 and STIP1 were significantly and persistently higher in T2D compared to normal controls for up to 24-h, suggesting that in T2D the heat shock and related proteins are preconditioned for an enhanced response. As the time course follow-up did not persist longer than 24-h, it is not known when these proteins reverted to baseline. The lack of a fall in T2D may indicate that chronic stress has primed the UPS (UBE2N, UBE2L3) to maintain its activity and levels in the face of this additional stress. Why the levels of UBE2N, UBE2L3 and STIP1 should fall in the control subjects in response to hypoglycemia, whilst UBE2G2 showed no apparent change in level, is unclear, and whether the loss of regulation of these specific and tightly controlled proteins would lead to a detrimental effect is unknown. The plasma measurement of these proteins in this study will be a surrogate for their activity and modulation at the cellular level. There is a wealth of data on heat shock and related protein cellular responses, and their regulation in vitro and in animal models, but there is a paucity of studies on their plasma levels following a time course of physiological stress in humans, and this study is the first in hypoglycemic stress. These questions need to be addressed to better understand diabetes complication development.

A significant increase in interleukin-6 was seen in both control and in T2D subjects at 4-h after induced hypoglycemia. IL-6 is both a pro-inflammatory cytokine in diabetes^47^ as well as an anti-inflammatory myokine^48^. IL-6 positively correlates with HSP70 (HSPA1A) in an anti-inflammatory response in traumatic brain injury^49^. In accord with that interaction, here IL-6 correlated with HSPAIA in controls alone, suggesting that it was associated with the HSP response and is perhaps dysregulated in T2D, though it cannot be excluded that the elevation in IL-6 was an independent effect in response to the hypoglycemia. In addition, the anti-inflammatory cytokine, IL-10, was decreased 2-h post-hypoglycemia in T2D only, lending further weight to the concept that the inflammatory response in T2D is dysregulated. That the other pro-inflammatory proteins were not increased is more in keeping with IL-6 acting in concert with heat shock and related proteins affording a protective effect, though others have suggested that the IL-6 response in hypoglycemia is part of a pro-inflammatory and pro-atherothrombotic response^50^. Intracellular HSPs (iHSPs) are critical in prevention of cell stress^51^ and it has been shown that, in T2D, iHSP levels are reduced, possibly because the cell has reduced ability to export them extracellularly through the secretory process^51^. Increased extracellular HSPs can promote oxidative damage and are linked to pro-inflammatory pathways in T2D^52^. HSPD1 (HSP60), that increased at 2-h only in controls, has been associated with renal tubular dysfunction in diabetes through modulation of oxidative stress in accord with the functions of other HSPs^53^.

Duration of diabetes in this T2D population was relatively short and therefore it would be important to repeat this study in a cohort where disease duration was longer, and in those with and without diabetes-related complications. Furthermore, it would be of importance to determine the heat shock and related protein response to less severe or more prolonged hypoglycemia and to determine when changes returned to baseline beyond 24-h. Of note, the changes seen here in the heat shock and related proteins may be due to both the hypoglycemic stress as well as the counter-regulatory response to hypoglycemia.

The group of T2D subjects of short disease duration enrolled in this study who were relatively treatment naïve is a strength of the study. The major study limitations are the small study numbers as a larger population may have highlighted further heat shock and related protein changes over the time course. However, it would be anticipated that the severe hypoglycemic episode induced would effect apparent changes in protein levels. Though the T2D subjects were older and more obese, this should not have altered the expression of these proteins to the hypoglycemic insult. As all the subjects were Caucasian, the results may not be generalizable to other ethnic groups. Given the changes in heat shock and related proteins in the controls at 24-h, in future studies it would be of interest to lengthen this period to determine when the changes revert to baseline. It should also be noted that the serum measurement of the individual heat shock and related proteins may not reflect their levels or activity at the cellular level.

In conclusion, heat shock and related proteins differed at baseline between T2D and controls with an exaggerated response of heat shock and related proteins to hypoglycemia that returned to baseline, though with changes in heat shock and related proteins at 24-h, where an increase in pro-inflammatory interleukin-6 was seen, suggesting that the heat shock and related protein system is overactivated due to underlying inflammation and oxidative stress in T2D.

## Supplementary Information

Supplementary Information.

## Data Availability

All the data for this study will be made available upon reasonable request to the corresponding author.

## Abbreviations

T2D: Type 2 diabetes
BMI: Body mass index
REC: Research ethics committee
IAPP: Islet amyloid polypeptide
ER: Endoplasmic reticulum
SOMA: Slow Off-rate Modified Aptamer
HSPs: Heat shock proteins
UPS: Ubiquitin proteasome system
UPR: Unfolded protein response
HSP70: Heat shock protein 70
HSP27: Heat shock protein 27
HSP90: Heat shock protein 90
HSPA1A: Heat shock 70 kDa protein 1A
HSPA8: Heat shock cognate 71 kDa protein
HSPB1: Heat shock protein beta-1
HSPD1: 60 KDa heat shock protein, mitochondrial
AIMP1: Aminoacyl tRNA synthase complex-interacting multifunctional protein 1
CDC37: Hsp90 co-chaperone Cdc37
CLU: Clusterin
DNAJB1: DnaJ homolog subfamily B member 1
MAPKAPK2: MAP kinase-activated protein kinase 2
MAPKAPK5: MAP kinase-activated protein kinase 5
PPID: Peptidyl-prolyl *cis*–*trans* isomerase D
PPP3CA: Serine/threonine-protein phosphatase 2B catalytic subunit alpha isoform
STIP1: Stress-induced-phosphoprotein 1
TLR4: Toll-like receptor 4
TLR4:MD-2 complex: Toll-like receptor 4 in complex with MD-2
CD274: Programmed cell death 1 ligand 1
EPHA2: Ephrin type-A receptor 2
SMAD3: Mothers against decapentaplegic homolog 3
E1: Ubiquitin activating enzyme
E2: Ubiquitin conjugating enzyme 2
UBE2G2: Ubiquitin-conjugating enzyme E2 G2
E3: Ubiquitin protein ligase
UBE2L3: Ubiquitin-conjugating enzyme
UBE2N: Ubiquitin-conjugating enzyme E2 N
UCHL1: Ubiquitin carboxyl-terminal hydrolase isozyme L1
STUB1: E3 ubiquitin-protein ligase CHIP
NFκB: Nuclear factor kappa-light-chain-enhancer of activated B cells
AKT: Protein kinase B
HSF: Heat shock factors
SNO: S-Nitrosylation
P38 MAPK: P38 mitogen-activated protein kinases
Bcl-xL: B-cell lymphoma-extra large
LRP1: Low density lipoprotein receptor-related protein 1
TGFR: Transforming growth factor beta receptors
IR: Insulin receptor
IRS1: Insulin receptor substrate 1
IL-6: Interleukin 6
IL-1α: Interleukin 1 alpha
IL-12: Interleukin 12
IFNγ: Interferon gamma
TNFα: Tumour necrosis factor alpha
