# Supplementary material for: Impact of severe hypoglycemia on the heat shock and related protein response
Source: Sci Rep. 2021 Aug 23;11:17057. doi: 10.1038/s41598-021-96642-8 (PMC8382834; doi:10.1038/s41598-021-96642-8)
Supplement: Supplementary file 1 — Supplementary Information. [file 41598_2021_96642_MOESM1_ESM.pdf]

Impact of Severe Hypoglycemia on the Heat Shock and Related Protein Response

Alexander S. Atkin[1@], Abu Saleh Md Moin, PhD[2@], Manjula Nandakumar PhD[2], Ahmed Al-Qaissi MD[3,4], Thozhukat Sathyapalan MD[3], Stephen L. Atkin MD[5*], Alexandra E. Butler MD[2*]

[1]Trinity College, Cambridge University, UK

[2] Diabetes Research Center (DRC), Qatar Biomedical Research Institute (QBRI), Hamad Bin Khalifa University (HBKU), Qatar Foundation (QF), PO Box 34110, Doha, Qatar

[3] Academic Endocrinology, Diabetes and Metabolism, Hull York Medical School, Hull, UK.

[4] Leeds Medical School, Leeds, UK

[5] Royal College of Surgeons of Ireland, Bahrain

@ joint first authors

*joint senior authors

Supplementary table 1. Demographic and clinical characteristics of the study participants. Data are presented as mean ± SD.

**Baseline**	**Type 2 Diabetes (n=23)**	**Controls (n=23)**	**p-value**
Age (years)	64±8	60±10	<0.0001
Sex (M/F)	12/11	11/12	0.77
Weight (kg)	90.9±11.1	79.5±8.8	<0.0001
Height (cm)	167±14	169±5	0.64
BMI (kg/m^2)	32±4	28±3	<0.0001
Systolic BP (mmHg)	132±8	122±8	0.001
Diastolic BP (mmHg)	81±7	75±6	0.003
Duration of diabetes (years)	4.5±2.2	N/A	
HbA1c (mmol/mol)	51.2±11.4	37.2±2.2	<0.0001
HbA1c (%)	6.8±1.0	5.6±0.2	<0.0001
Total cholesterol (mmol/l)	4.2±1.0	4.8±0.77	0.014
Triglyceride (mmol/l)	1.7±0.7	1.34±0.6	0.055
HDL-cholesterol (mmol/l)	1.1±0.3	1.5±0.4	0.001
LDL-cholesterol (mmol/l)	2.23±0.8	2.7±0.87	0.051
CRP (mg/l)	3.0±2.7	5.1±10.3	0.33

BMI: Body mass index, BP: Blood pressure, HDL-cholesterol: High density lipoprotein cholesterol, LDL-cholesterol: Low density lipoprotein cholesterol, CRP: C-reactive protein. HbA1c: Hemoglobin A1c

Supplementary table 2. Student's t-test values comparing protein levels for baseline versus hypoglycaemia and baseline versus 24-hours post-hypoglycemia in type 2 diabetes (T2D) and control subjects for the twenty-six proteins included in the analysis. HSP = heat shock protein.

Abbreviation		T2D vs Control (Baseline)	Baseline vs Hypoglycaemia		Baseline vs 24 hours	
			Control p-value	T2D p-value	Control p value	T2D p value
HSP90AA1	HSP90 alpha	0.44	0.88	0.95	0.72	0.85
HSP90AB1	HSP90 beta	0.5	0.26	0.59	0.12	0.65
HSPA1A	Heat shock 70 kDa protein 1A	0.27	**0.02**	0.07	0.91	0.32
HSPA8	Heat shock cognate 71 kDa protein	0.20	0.06	0.78	0.51	0.51
HSPB1	Heat shock protein beta-1	0.37	**0.015**	0.10	0.48	0.9
HSPD1	60 kDa heat shock protein, mitochondrial	0.74	0.72	0.33	0.50	0.40
AIMP1	Aminoacyl tRNA synthase complex-interacting multifunctional protein 1	0.10	0.07	0.60	0.96	0.09
CDC37	Hsp90 co-chaperone Cdc37	0.36	0.09	0.58	0.11	0.34
CLU	Clusterin	0.16	0.88	0.76	0.69	0.72
DNAJB1	DnaJ homolog subfamily B member 1	0.80	0.08	0.23	0.38	0.60
MAPKAPK2	MAP kinase-activated protein kinase 2	0.65	0.37	0.64	0.67	0.50

MAPKAPK5	MAP kinase-activated protein kinase 5	**0.04**	**0.04**	0.27	0.83	0.07
PPID	Peptidyl-prolyl cis-trans isomerase D	0.24	0.17	0.70	0.94	0.94
PPP3CA	Serine/threonine-protein phosphatase 2B catalytic subunit alpha isoform	0.28	0.72	0.056	0.25	0.27
STIP1	Stress-induced-phosphoprotein 1	0.57	0.10	0.66	**0.006**	0.09
STUB1	E3 ubiquitin-protein ligase CHIP	**0.01**	0.78	0.60	0.48	0.57
TLR4	Toll-like receptor 4	0.30	0.76	0.33	0.82	0.40
TLR4:MD-2 complex	Toll-like receptor 4 in complex with MD-2	0.48	0.05	0.72	0.52	0.19
HSP 90a/b	HSP90 dimer	0.20	0.70	0.57	0.44	0.35
CD274	Programmed cell death 1 ligand 1	0.54	0.97	0.88	0.98	0.90
EPHA2	Ephrin type-A receptor 2	0.82	0.32	**0.03**	0.50	0.07
SMAD3	Mothers against decapentaplegic homolog 3	0.88	**0.008**	0.75	0.29	0.39
UBE2G2	Ubiquitin-conjugating enzyme E2 G2	**0.006**	0.83	0.90	0.76	0.50
UBE2L3	Ubiquitin-conjugating enzyme E2L 3	0.66	0.09	0.37	**0.04**	0.27

UBE2N	Ubiquitin-conjugating enzyme E2 N	0.80	0.07	0.21	**0.006**	0.09
UCHL1	Ubiquitin carboxyl-terminal hydrolase isozyme L1	0.46	0.**37**	0.73	0.69	0.30

Supplementary Figure 1

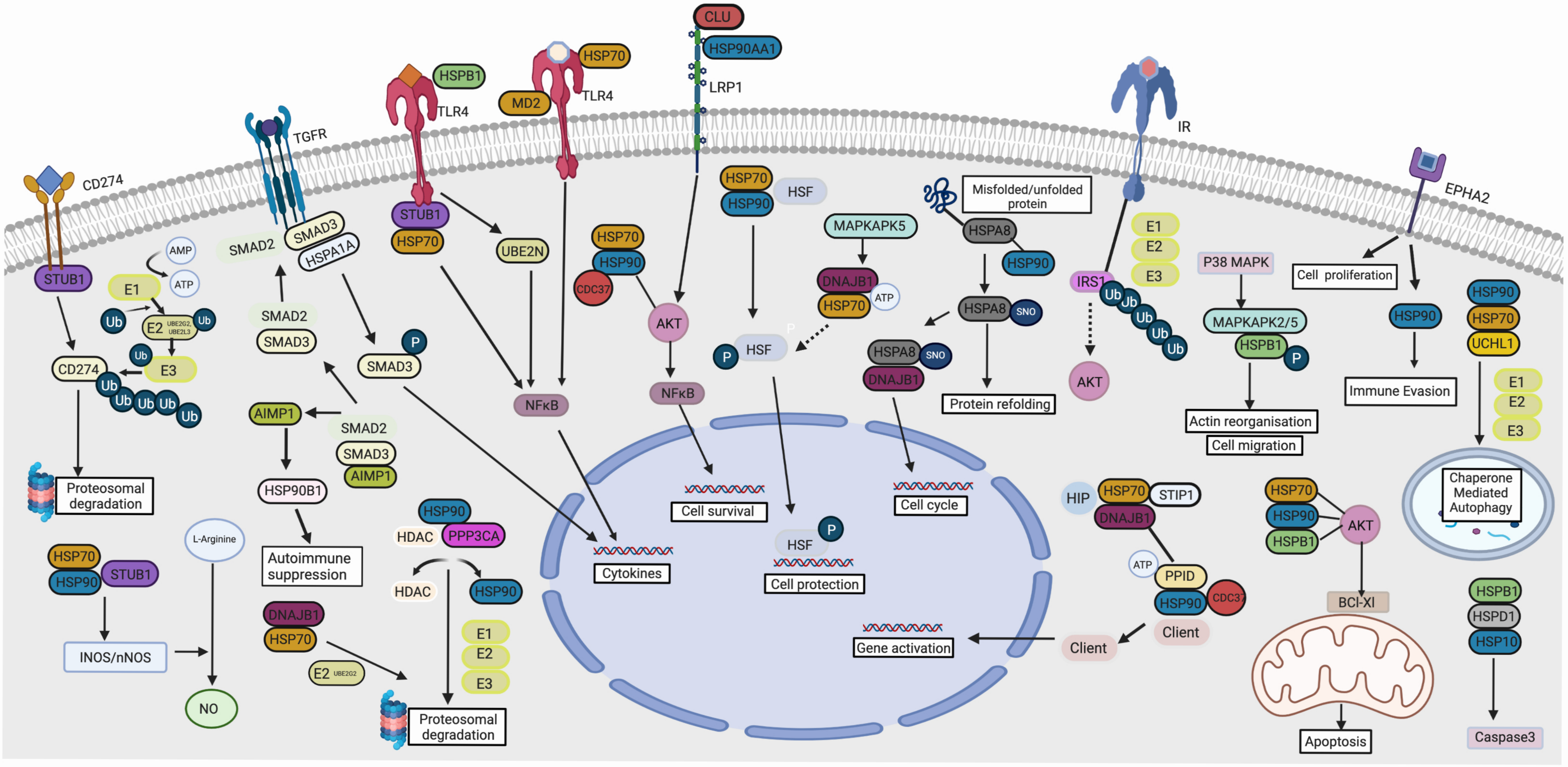

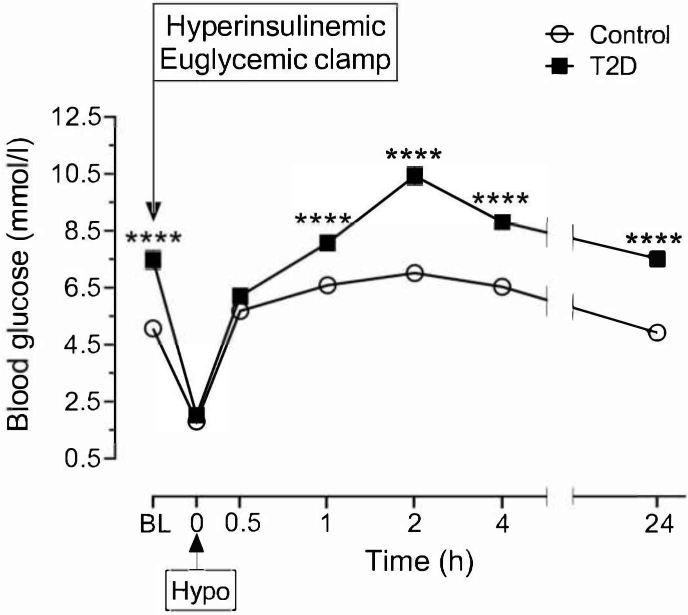

Supplementary Figure 2

Supplementary figure 3. Proteins that did not differ either between T2D and control subjects or during the post hypoglycemia time course.

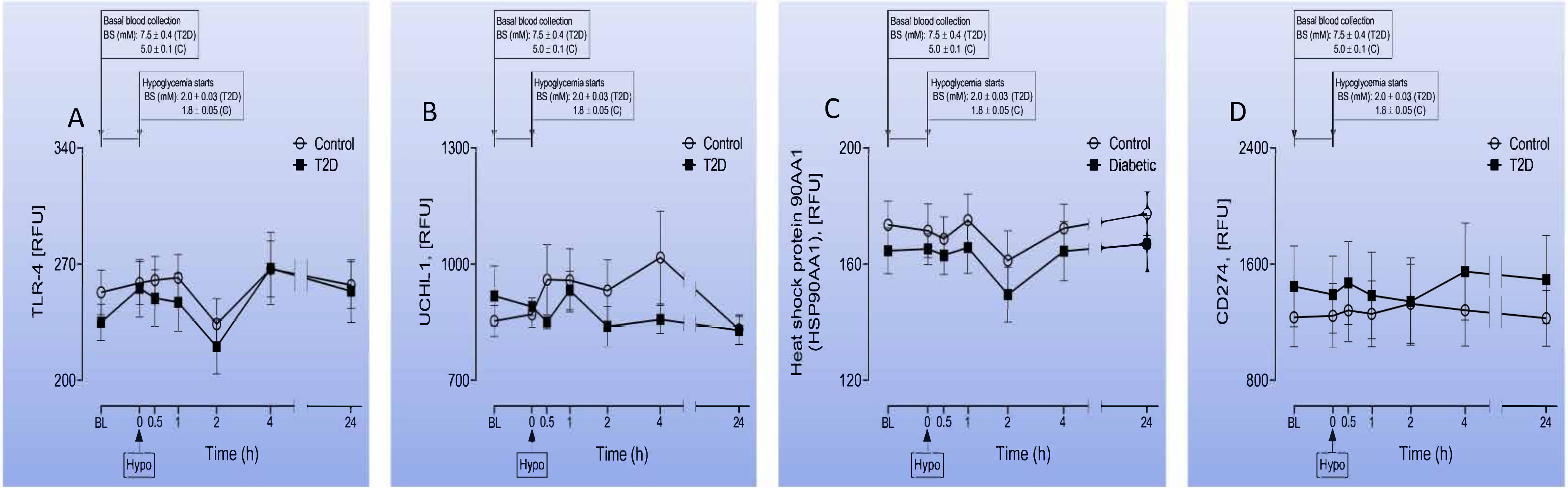

Supplementary figure 4

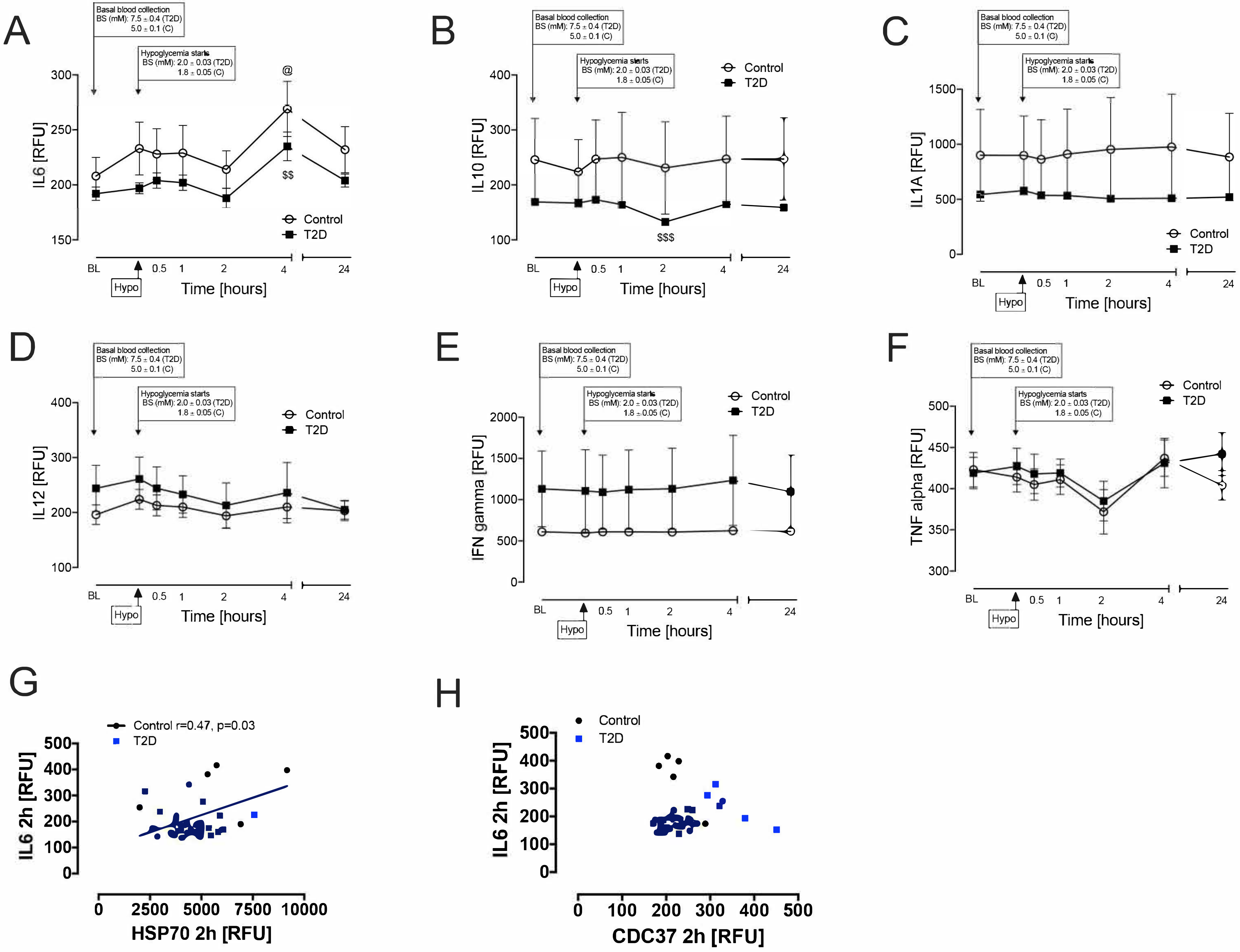

Supplementary figure legends

Supplementary Figure 1. Schematic figure showing an overview of interactions between HSP and associated proteins that are differentially expressed in response to hypoglycemia. These interactions decide the fate of the downstream signaling pathway. The HSP and associated proteins interact with the cell surface receptors and or with each other in response to different stimuli, including accumulated unfolded/misfolded proteins, hormones and cellular/environmental stress and regulate different molecules affecting a spectrum of biological functions such as apoptosis, autophagy, cell migration and alterations in the immune response. The pathways depicted in the figure are the general pathways of HSP related signaling and are not restricted to a certain tissue or cell type.

Schematic created using Biorender (https://biorender.com)

HSP90 alpha (HSP90AA1, HSP90AB1, HSP90 beta, HSP 90a/b HSP90 dimer); HSPA1A, Heat shock 70 kDa protein 1A; HSPA8 Heat shock cognate 71 kDa protein ; HSPB1 Heat shock protein beta-1; HSPD1, 60 kDa heat shock protein, mitochondrial; AIMP1, Aminoacyl tRNA synthase complex-interacting multifunctional protein 1; CDC37 Hsp90 co-chaperone Cdc37; CLU, Clusterin; DNAJB1, DnaJ homolog subfamily B member 1; MAPKAPK2, MAP kinase-activated protein kinase 2; MAPKAPK5, MAP kinase-activated protein kinase 5; PPID, Peptidyl-prolyl cis-trans isomerase D; PPP3CA, Serine/threonine-protein phosphatase 2B catalytic subunit alpha isoform; STIP1, Stress-induced-phosphoprotein 1; TLR4, Toll-like receptor 4; TLR4:MD-2 complex, Toll-like receptor 4 in complex with MD-2; CD274, Programmed cell death 1 ligand 1; EPHA2, Ephrin type-A receptor 2; SMAD3, Mothers against decapentaplegic homolog 3; E1, Ubiquitin activating enzyme; E2, Ubiquitin conjugating enzymes 2 (UBE2G2, Ubiquitin-conjugating enzyme E2 G2; UBE2L3, Ubiquitin-conjugating enzyme; UBE2N,Ubiquitin-conjugating enzyme E2 N); UCHL1, Ubiquitin carboxyl-terminal hydrolase isozyme L1; E3, Ubiquitin ligases; STUB1, E3 ubiquitin-protein ligase CHIP; NFκB, nuclear factor kappa-light-chain-enhancer of activated B cells; AKT,

Protein kinase B, HSF, heat shock factors; SNO, S-Nitrosylation; P38 MAPK, p38 mitogen-activated protein kinases; Bcl-xL, B-cell lymphoma-extra large; LRP1, Low density lipoprotein receptor-related protein 1; TGFR, Transforming growth factor beta receptors; IR, Insulin receptor; IRS1, Insulin receptor substrate 1.

Supplementary Figure 2. **The comparison of blood glucose levels at baseline, at hypoglycaemia and post-hypoglycaemia up to 24 h**. Blood sampling was performed at baseline (BL), at hypoglycaemia (0 min) and post-hypoglycaemia (0.5, 1, 2, 4 and 24 h) for controls (white circles) and for type 2 diabetes (T2D) (black squares). At BL, blood sugar (BS) was 7.5 ± 0.4 mmol/l (for T2D) and 5.0 ± 0.1 mmol/l (for control, C). Insulin was infused (at a rate of 2 mU/ml/Kg body weight) by hyperinsulinemic euglycemic clamp. At hypoglycemia, BS was 2.0 ± 0.03 mmol/l (for T2D) and 1.8 ± 0.05 mmol/l (for controls).

Supplementary Figure 3. **Circulatory HSP and related proteins that did not differ with hypoglycemia or between T2D and controls.** Proteomic (Somalogic) analysis was undertaken to determine the plasma levels of HSP related proteins, Toll-like receptor 4 (TLR4) (**A**), Ubiquitin carboxyl-terminal hydrolase isozyme L1 (UCHL1) (**B**), Heat shock protein 90AA1 (HSP90A1A) (**C**), Programmed cell death 1 ligand 1 (CD274) (**D**) at baseline (BL) during and after iatrogenic induction of hypoglycemia for control (C) and type 2 diabetes (T2D) subjects. Blood sampling was performed at BL, at hypoglycemia (0 min) and post-hypoglycemia (0.5-hour, 1-hour, 2-hours, 4-hours and 24-hours) for controls (white circles) and for T2D (black squares).

Supplementary Figure 4. **Changes of circulatory pro-inflammatory and anti-inflammatory cytokines in response to hypoglycemia in control subjects and subjects with**

T2D. Proteomic (Somalogic) analysis was undertaken to determine the plasma levels of HSP related proteins, Interleukin 6 (IL-6) (**A**), Interleukin 10 (IL-10) (**B**), Interleukin 1A (IL-1A) (**C**), Interleukin 12 (IL-12) (**D**), Interferon gamma (IFN gamma) (**E**), TNF alpha (TNF alpha) (**F**) at baseline (BL), during and after iatrogenic induction of hypoglycemia for control (C) and type 2 diabetes (T2D) subjects. Blood sampling was performed at BL, at hypoglycemia (0 min) and post-hypoglycemia (0.5-hour, 1-hour, 2-hours, 4-hours and 24-hours) for controls (white circles) and for T2D (black squares). Correlations of plasma levels of IL-6 at 2-hours post-hypoglycemia with HSP70 (**G**) and CDC37 (**H**).
